# Supplementary material for: Structural Investigation of Magnesium Complexes Supported by a Thiopyridyl Scorpionate Ligand
Source: Molecules. 2022 Jul 18;27(14):4564. doi: 10.3390/molecules27144564 (PMC9318590; doi:10.3390/molecules27144564)
Supplement: Supplementary file 1 [file molecules-27-04564-s001.zip › molecules-1807774-supplementary.pdf]

Structural Investigation of Magnesium Complexes Supported by a Thiopyridyl Scorpionate Ligand

Matthew P. Stevens [1], Emily Spray [1], Iñigo J. Vitorica-Yrezabal [2], Kuldip Singh [1], Vanessa M. Timmermann [1], Lia Sotorrios [3,*] and Fabrizio Ortu [1,*]

[1] School of Chemistry, University of Leicester, University Road, Leicester LE1 7RH, UK; mps28@leicester.ac.uk (M.P.S.); emily.spray@hotmail.co.uk (E.S.); ks42@leicester.ac.uk (K.S.); vanessa.timmermann@siigroup.com (V.M.T.)

[2] Department of Chemistry, The University of Manchester, Oxford Road, Manchester M13 9PL, UK;
inigo.vitorica@manchester.ac.uk

[3] Institute of Chemical Sciences, Heriot-Watt University, Edinburgh EH14 4AS, UK

* Correspondence: l.sotorrios@hw.ac.uk (L.S.); fabrizio.ortu@leicester.ac.uk (F.O.); Tel.: +44-(0)116-294-4670 (F.O.)

S1. NMR data **S2**
S2. IR data **S10**
S3. Crystallography **S12**
S4. Computational data **S16**

S1. NMR data

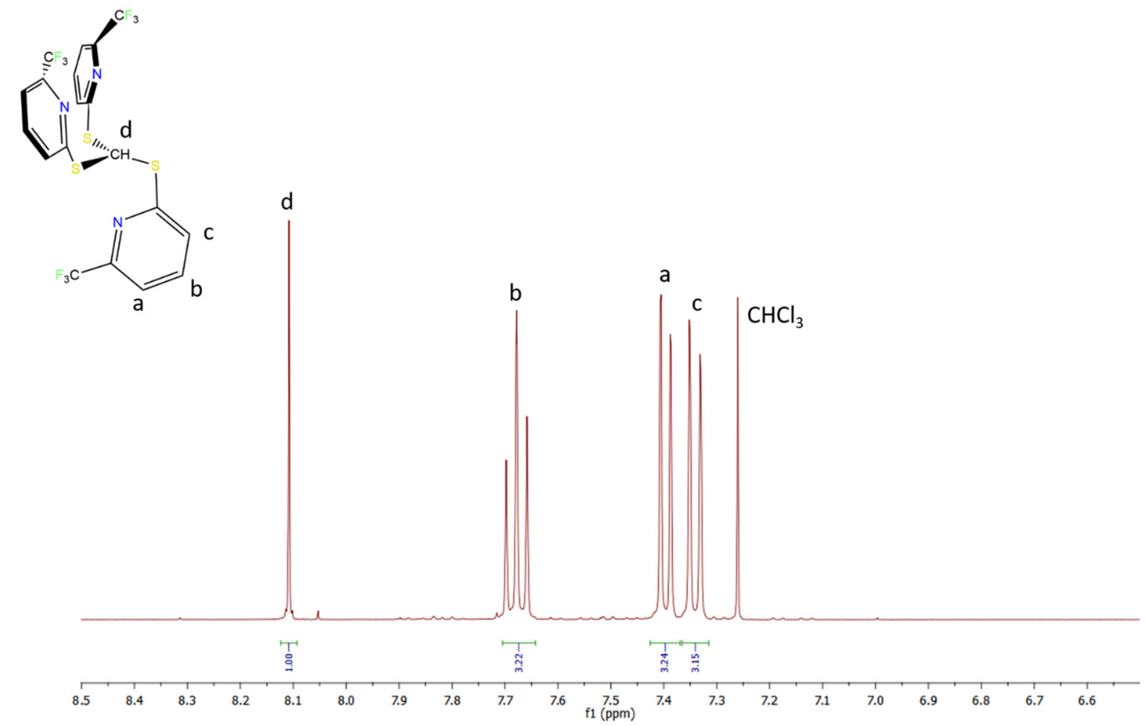

Figure S1: ^{1}H NMR (400 MHz, 298 K, $CDCl_3$) spectrum of $HTptm^{CF3}$, with assignment.

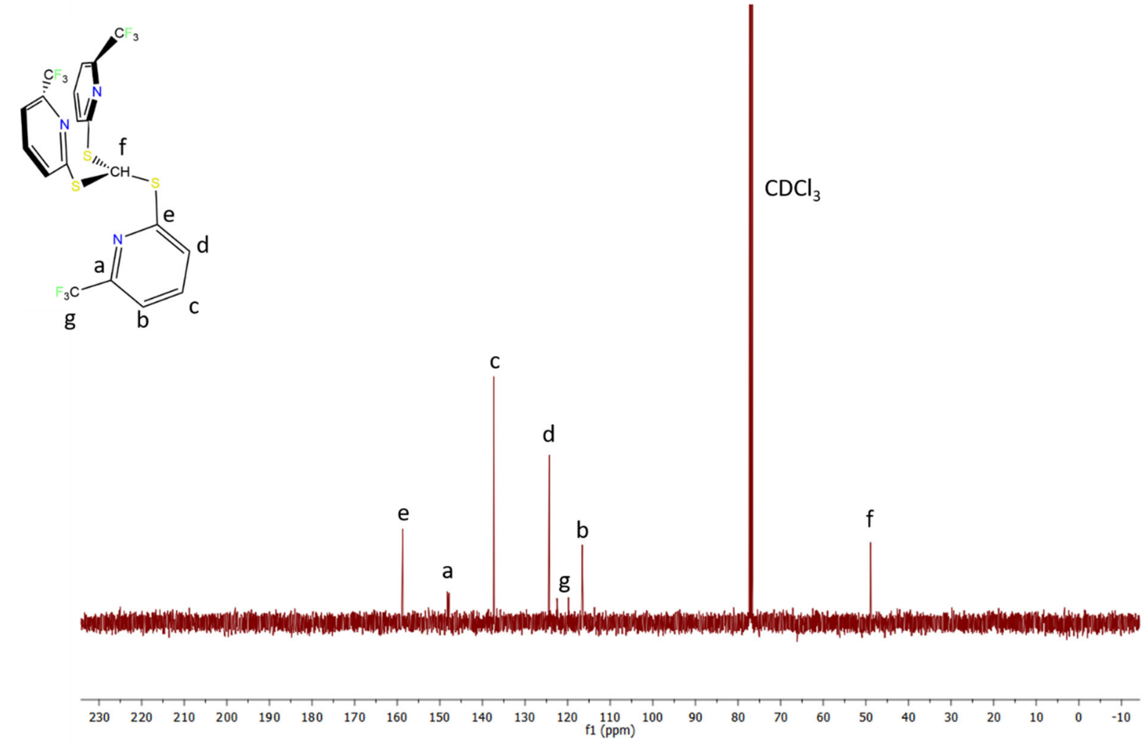

Figure S2: $^{13}C\{^{1}H\}$ NMR (100 MHz, 298 K, $CDCl_3$) spectrum of $HTptm^{CF3}$, with assignment.

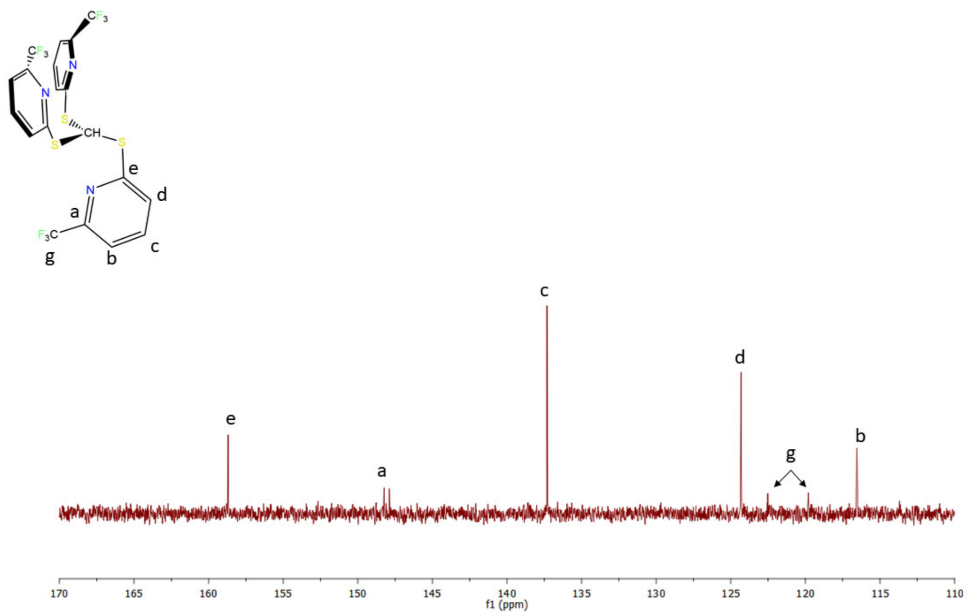

Figure S3: $^{13}C\{^{1}H\}$ NMR (100 MHz, 298 K, $CDCl_3$) spectrum of $HTptm^{CF3}$ in the region 110–170 ppm, with assignment.

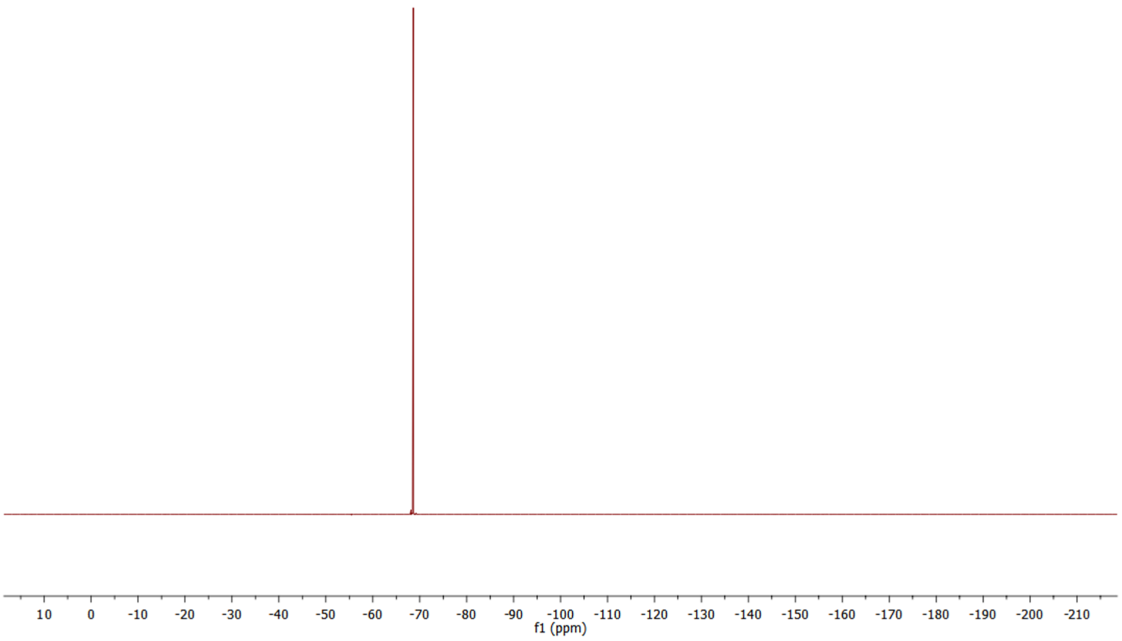

Figure S4: $^{19}F\{^{1}H\}$ NMR (376 MHz, 298 K, $CDCl_3$) spectrum of $HTptm^{CF3}$.

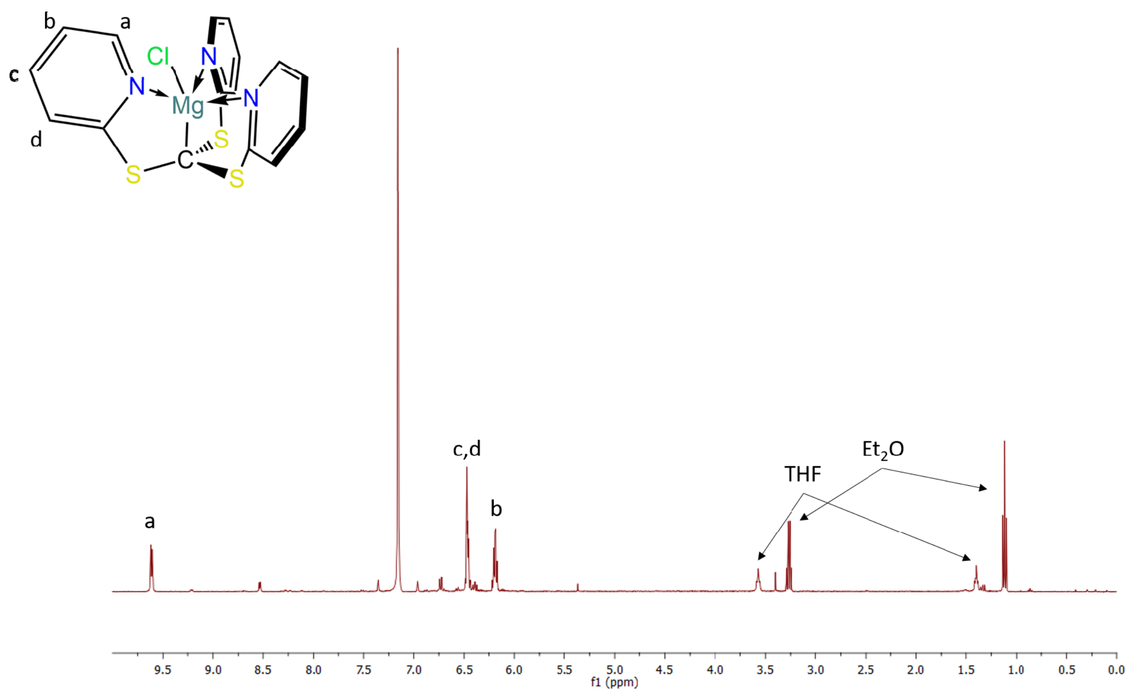

Figure S5: ^{1}H NMR (400 MHz, 298 K, C_6D_6) spectrum of **1-Cl**, with assignment.

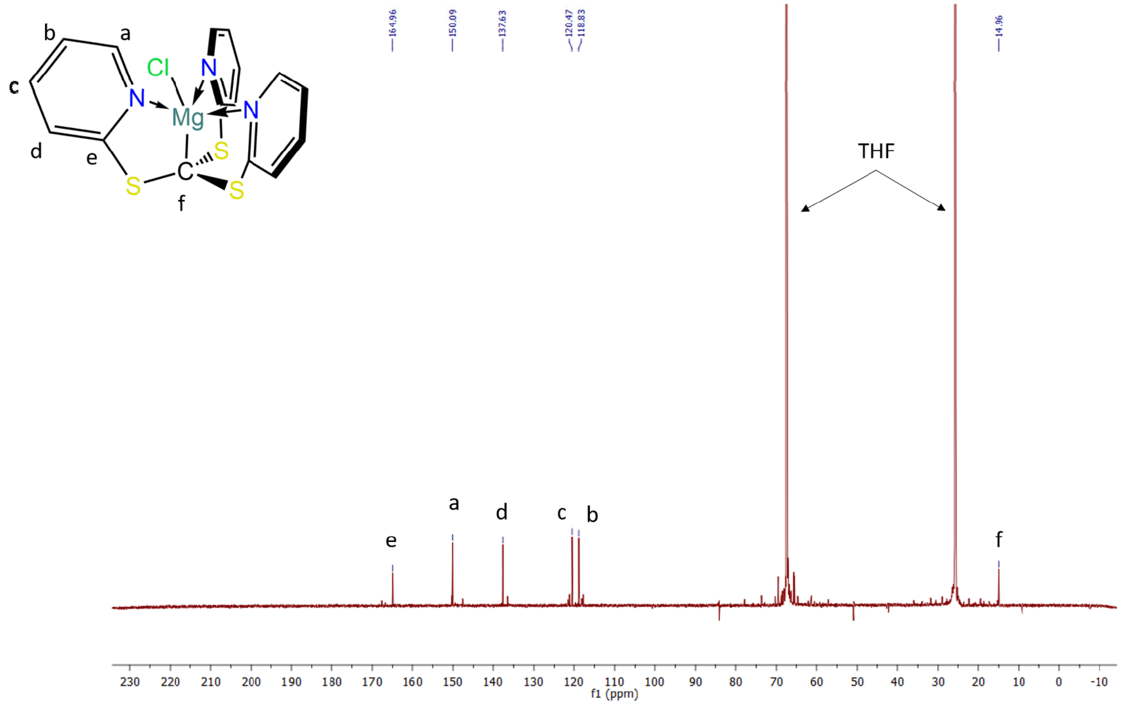

Figure S6: $^{13}C\{^{1}H\}$ NMR (125 MHz, 298 K, C_4D_8O) spectrum of **1-Cl**, with assignment.

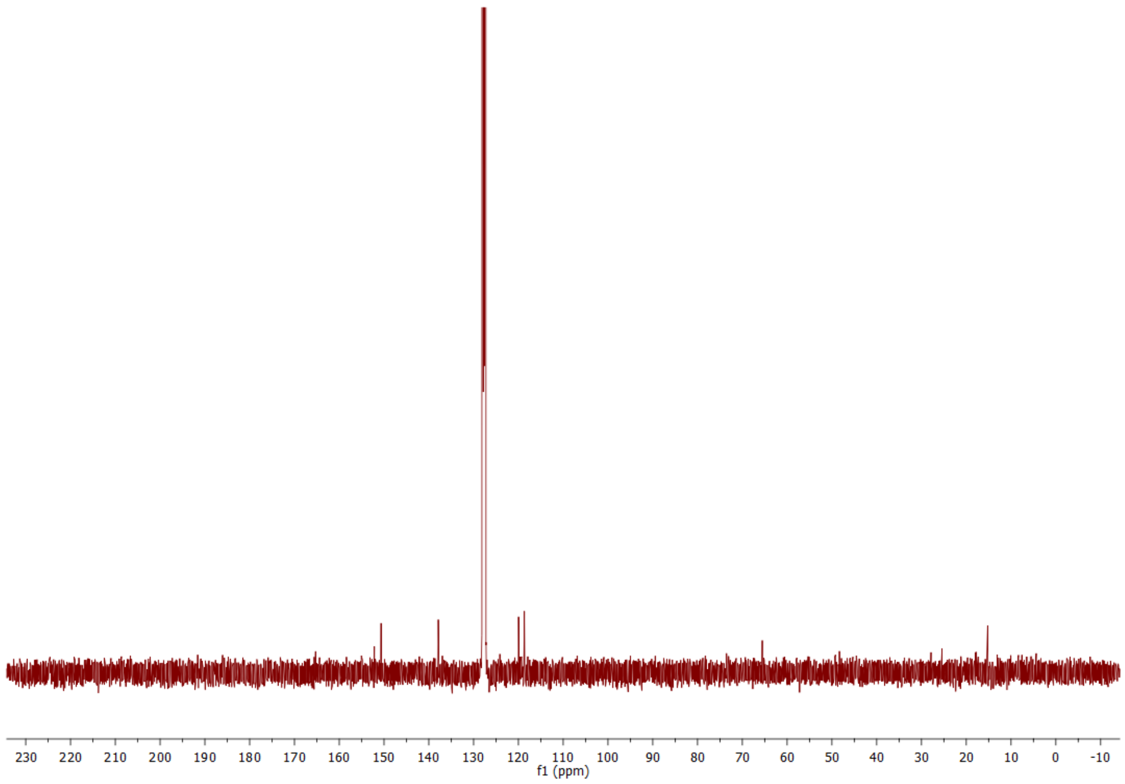

Figure S7: ${}^{13}C\{{}^{1}H\}$ NMR (100 MHz, 298 K, C_6D_6) spectrum of **1-Cl**.

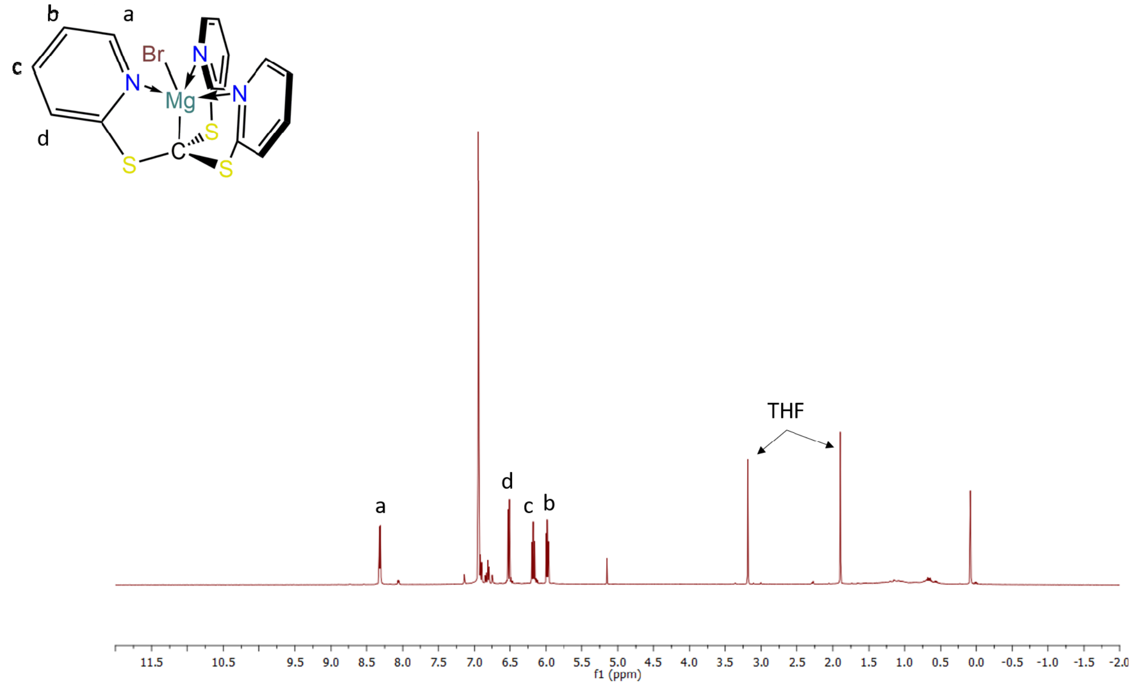

Figure S8: ${}^{1}H$ NMR (400 MHz, 298 K, C_6D_6) spectrum of **1-Br**, with assignment.

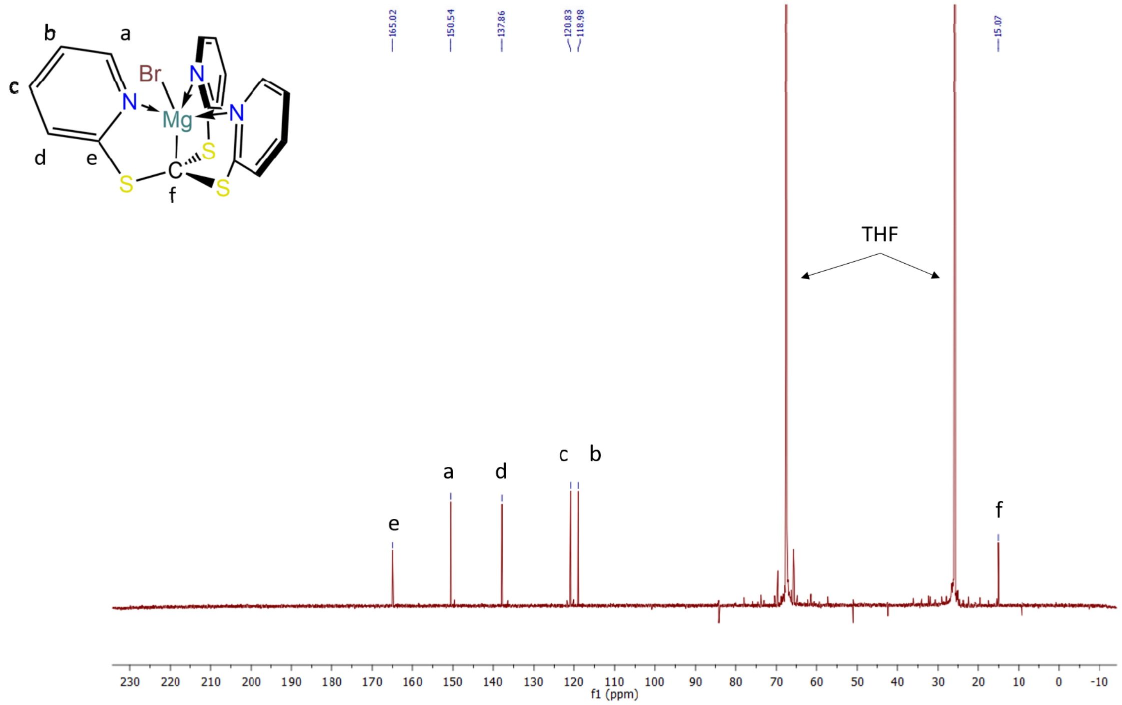

Figure S9: $^{13}C\{^{1}H\}$ NMR (125 MHz, 298 K, C_4D_8O) spectrum of **1-Br**, with assignment.

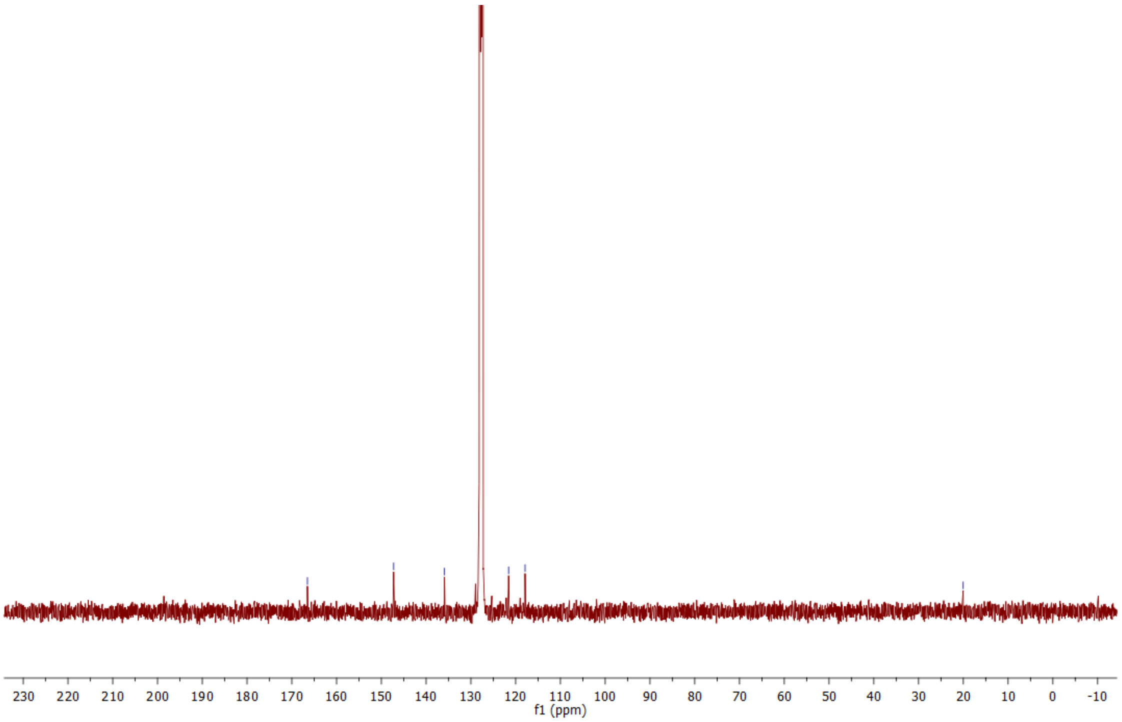

Figure S10: $^{13}C\{^{1}H\}$ NMR (100 MHz, 298 K, C_6D_6) spectrum of **1-Br**.

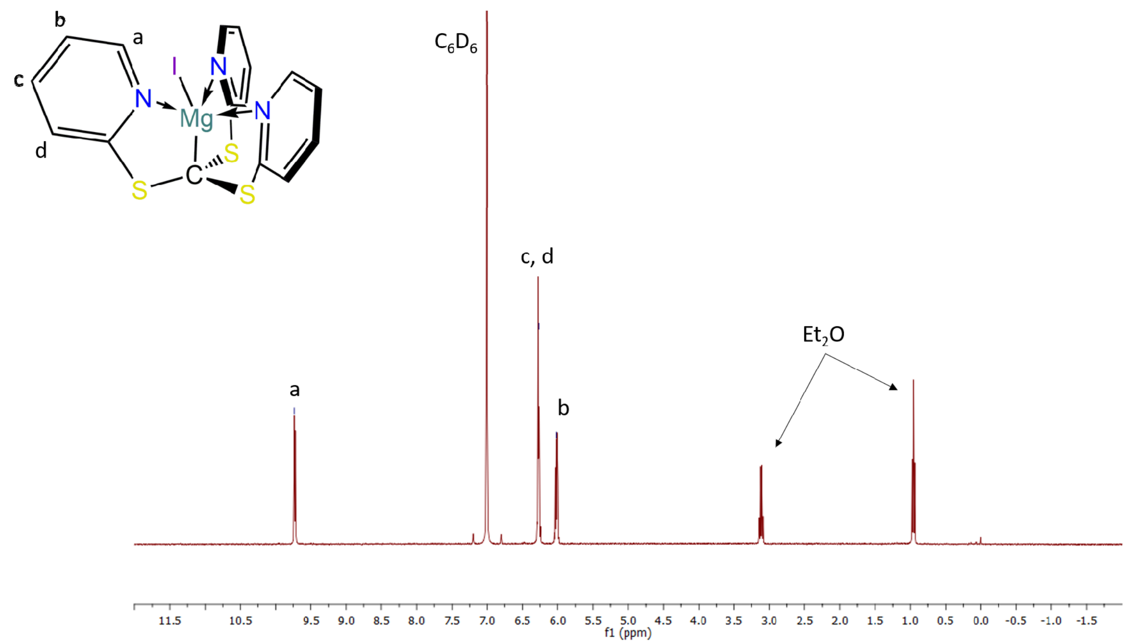

Figure S11: ^{1}H NMR (400 MHz, 298 K, C_6D_6) spectrum of **1-I**, with assignment.

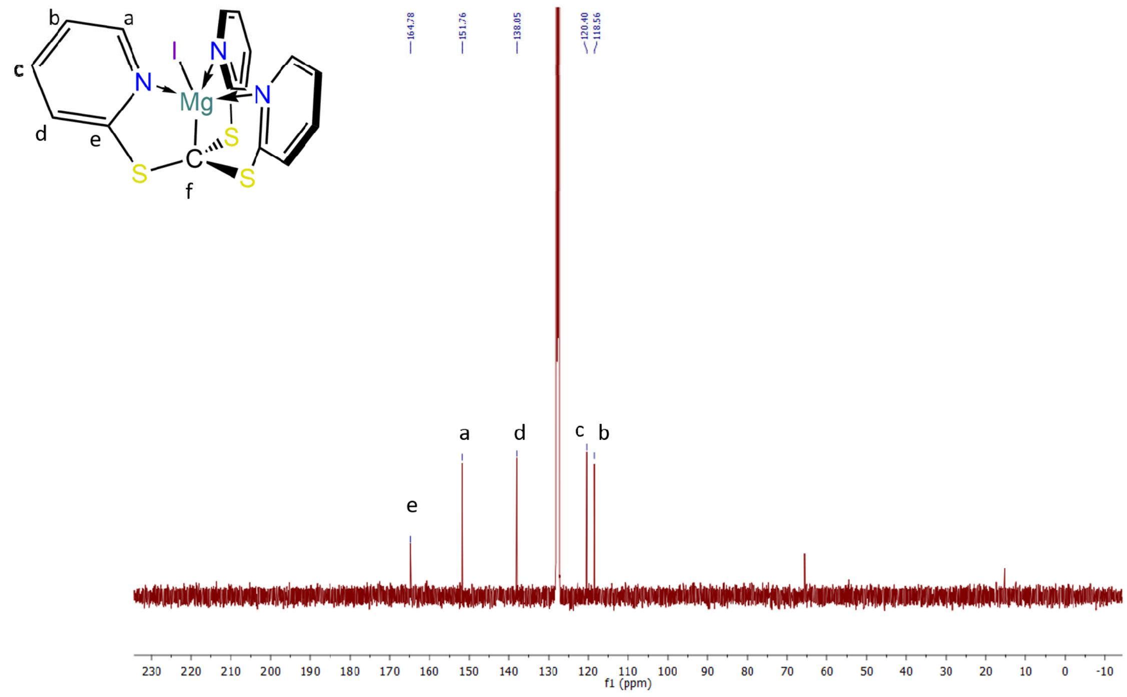

Figure S12: $^{13}C\{^{1}H\}$ NMR (100 MHz, 298 K, C_6D_6) spectrum of **1-I**, with assignment.

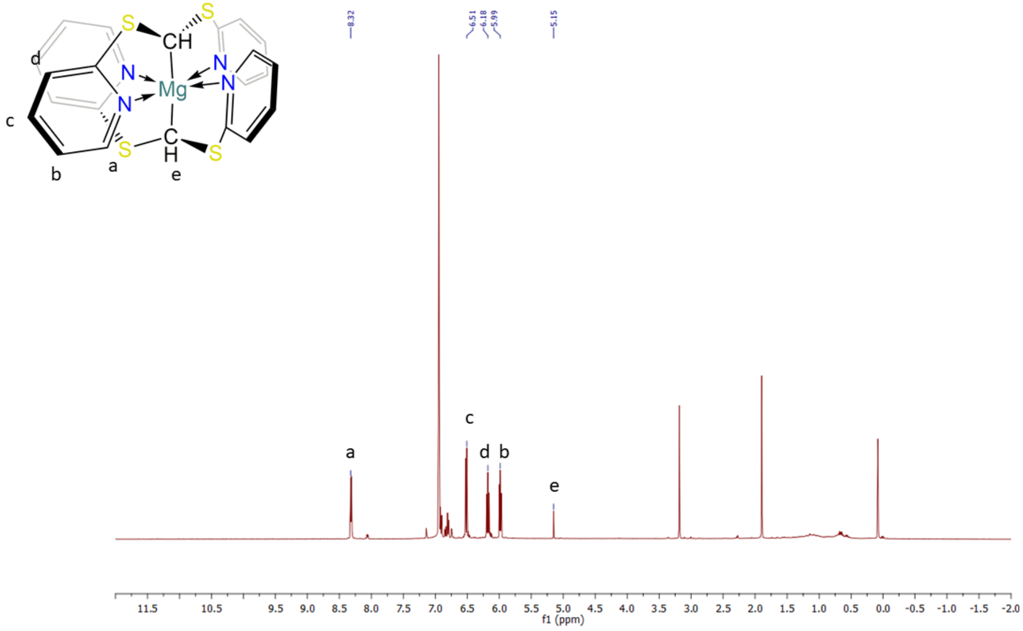

Figure S13: ^{1}H NMR (400 MHz, 298 K, C_6D_6) spectrum of **2**, with assignment.

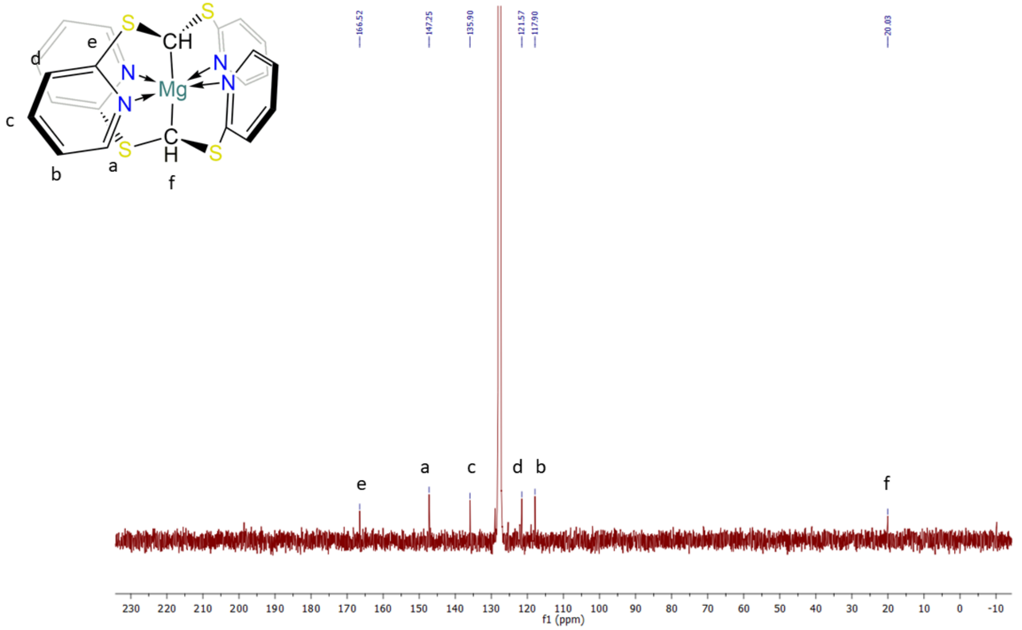

Figure S14: ^{13}C{^{1}H} NMR (100 MHz, 298 K, C_6D_6) spectrum of **2**, with assignment.

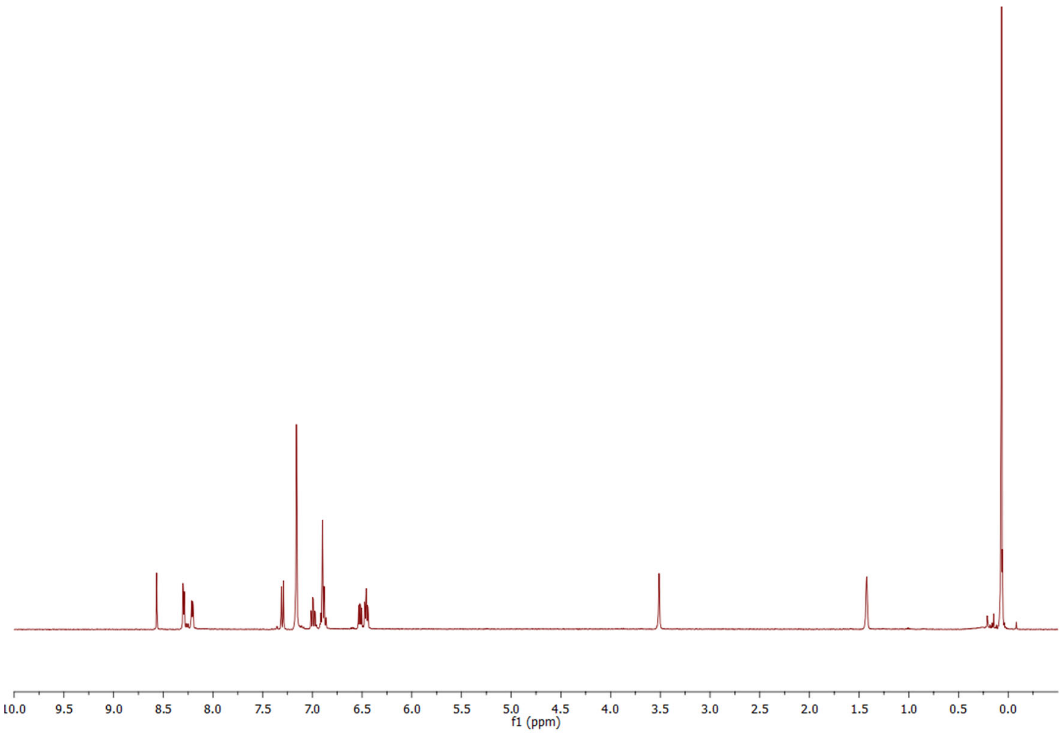

Figure S15: ^{1}H NMR (400 MHz, 298 K, C_6D_6/C_4D_8O) spectrum of the NMR scale reaction product of KN″ and HTptm.

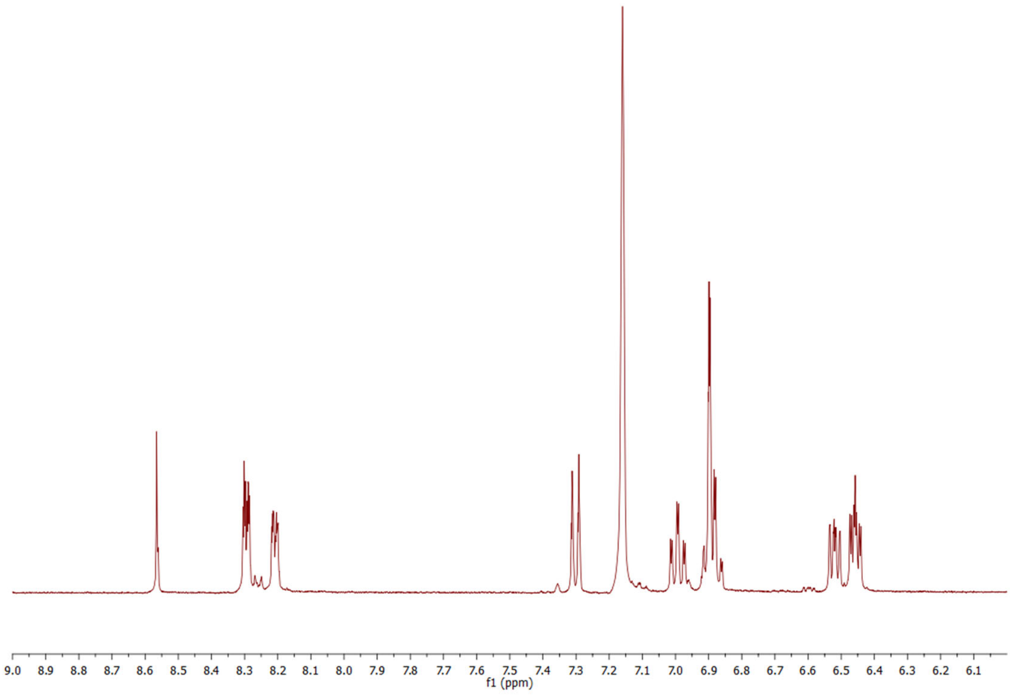

Figure S16: ^{1}H NMR (400 MHz, 298 K, C_6D_6/C_4D_8O) spectrum in the region 6.0-9.0 ppm of the NMR scale reaction product of KN″ and HTptm.

S2. IR data

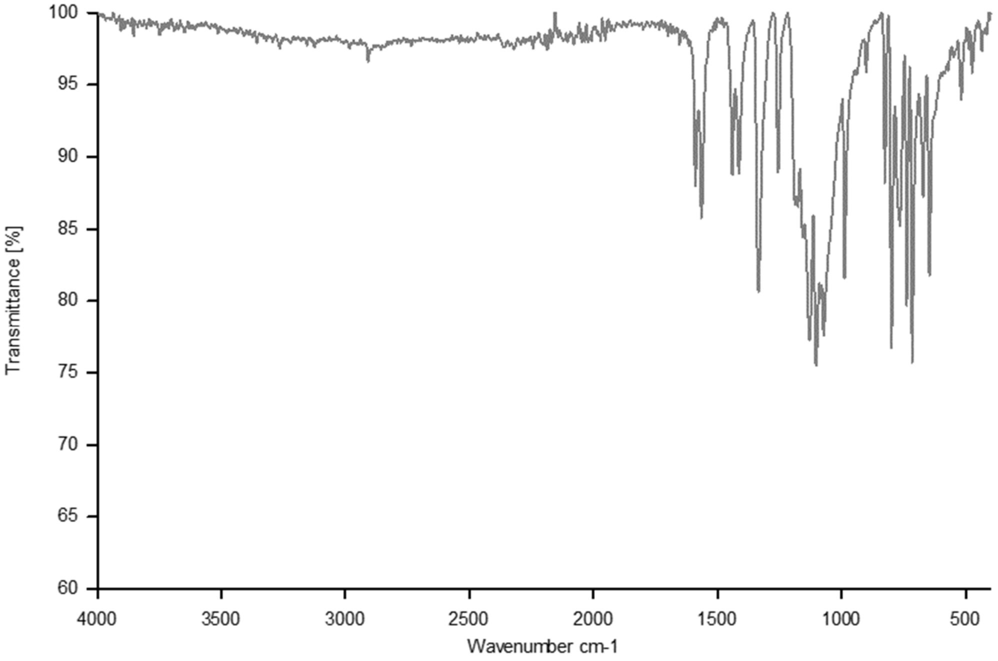

Figure S17: FTIR spectrum of $HTptm^{CF3}$.

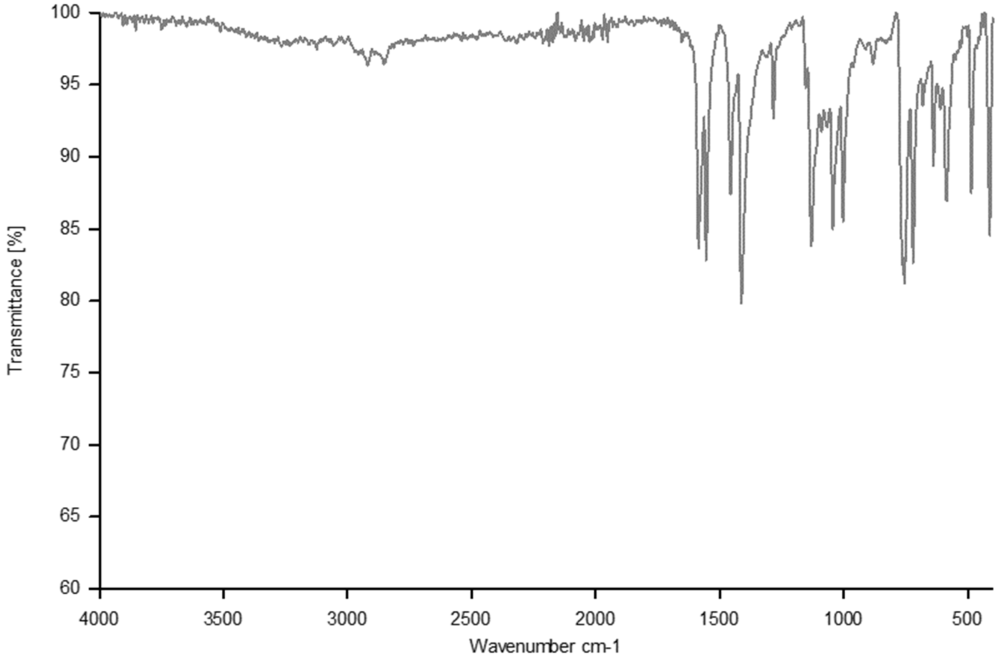

Figure S18: FTIR spectrum of **1-Cl**.

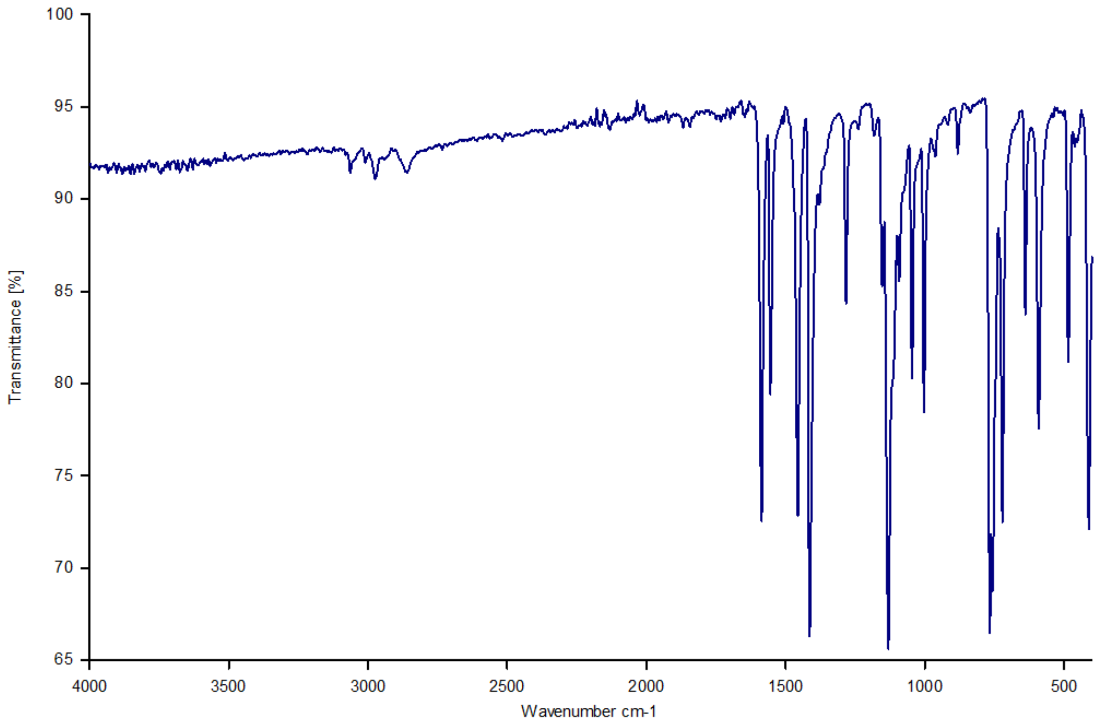

Figure S19: FTIR spectrum of **1-Br**.

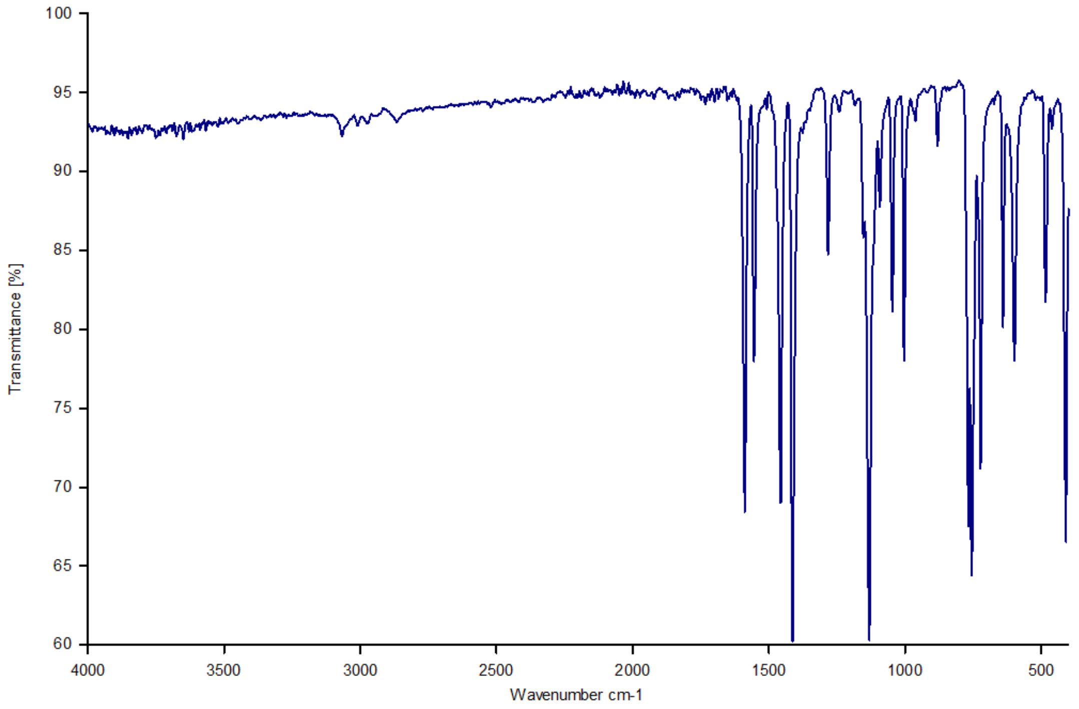

Figure S20: FTIR spectrum of **1-I**.

S3. Crystallography

Table S1: Crystallographic data for **1-X**

[a]Conventional $R = \Sigma||Fo| - |Fc||/\Sigma|Fo|$; $Rw = [\Sigma w(Fo^2 - Fc^2)^2/\Sigma w(Fo^2)^2]^{1/2}$; $S = [\Sigma w(Fo^2 - Fc^2)^2/\text{no. data} - \text{no. params})]^{1/2}$ for all data.

	**1-Cl**	**1-Br**	**1-I**
Formula	$C_{16}H_{12}ClMgN_3S_3$, C_3H_3	$2(C_{16}H_{12}BrMgN_3S_3)$, C_7H_8	$1.5(C_{32}H_{24}I_2Mg_2N_6S_6)$
Formula weight	441.28	985.50	1481.03
Crystal size, mm	0.03 × 0.06 × 0.12	0.05 × 0.08 × 0.25	0.04 × 0.09 × 0.49
Crystal system	monoclinic	triclinic	orthorhombic
Space group	$P2_1/c$	P-1	Pbcn
a, Å	12.5335(3)	9.7481(6)	9.7312(6)
b, Å	9.6427(3)	14.6381(10)	16.7573(9)
c, Å	16.7854(4)	16.5395(9)	34.9877(19)
α, °	90	109.917(6)	90
β, °	107.957(3)	91.018(5)	90
γ, °	90	107.003(6)	90
V, $Å^3$	1929.81(9)	2103.8(3)	5705.4(6)
Z	4	2	4
ρ_{calc}, g cm^3	1.519	1.556	1.724
μ, mm^{-1}	5.181	2.292	2.049
F(*000*)	908	996	2904
No. of reflections (unique)	3987 (3987)	14556 (7618)	38213 (5225)
S^a	1.22	1.03	1.05
$R_1(wR_2)$ ($F^2 > 2\sigma(F^2)$)	0.0533 (0.1774)	0.0895 (0.2455)	0.0758 (0.1970)
R_{int}	0.042	0.128	0.141
Min./max. diff map, $Å^{-3}$	-0.62, 0.89	-0.69, 0.88	-1.24, 0.80

Table S2: Crystallographic data for $HTptm^{CF3}$, **1-I•THF** and **2**

[a]Conventional $R = \Sigma||Fo| - |Fc||/\Sigma|Fo|$; $Rw = [\Sigma w(Fo^2 - Fc^2)^2/\Sigma w(Fo^2)^2]^{1/2}$; $S = [\Sigma w(Fo^2 - Fc^2)^2$/no. data – no. params)]$^{1/2}$ for all data.

	$HTptm^{CF3}$	**1-I•THF**	**2**
Formula	$C_{19}H_{10}F_9N_3S_3$	$C_{20}H_{20}IMgN_3OS_3$	$C_{22}H_{18}MgN_4S_4$, 0.5($C_{12}H_{12}$)
Formula weight	547.48	565.78	569.06
Crystal size, mm	0.12 x 0.27 x 0.31	0.20 × 0.36 × 0.49	0.22 × 0.31 × 0.40
Crystal system	monoclinic	monoclinic	triclinic
Space group	$P2_1/c$	$P2_1/n$	P-1
a, Å	8.0956(7)	10.0031(9)	8.8749(12)
b, Å	14.0932(12)	14.2808(14)	9.8170(14)
c, Å	19.8006(14)	16.5175(16)	9.9316(13)
α, °	90	90	106.175(12)
β, °	95.213(7)	89.941(9)	115.598(13)
γ, °	90	90	95.669(12)
V, $Å^3$	2249.8(3)	2359.6(4)	725.1(2)
Z	4	4	1
ρ_{calc}, g cm^3	1.616	1.593	1.303
μ, mm^{-1}	0.417	1.666	0.374
F(*000*)	1096	1128	296
No. of reflections (unique)	15077 (4117)	4145 (4145)	4879 (2540)
S^a	1.10	1.04	1.11
$R_1(wR_2)$ ($F^2 > 2\sigma(F^2)$)	0.0810 (0.1504)	0.1044 (0.2728)	0.0867 (0.2389)
R_{int}	0.094	0.151	0.041
Min./max. diff map, $Å^{-3}$	-0.27, 0.29	-1.41, 2.20	-0.40, 0.78

Table S3: Crystallographic data for $(C_5H_4N\text{-}S)_2C{=}C(C_5H_4N\text{-}S)_2$.

[a]Conventional $R = \Sigma||Fo| - |Fc||/\Sigma|Fo|$; $Rw = [\Sigma w(Fo^2 - Fc^2)^2/\Sigma w(Fo^2)^2]^{1/2}$; $S = [\Sigma w(Fo^2 - Fc^2)^2$/no. data – no. params)]$^{1/2}$ for all data.

	**$(C_5H_4N\text{-}S)_2C{=}C(C_5H_4N\text{-}S)_2$**
Formula	$C_{22}H_{16}N_4S_4$
Formula weight	464.63
Crystal size, mm	0.03 × 0.10 × 0.12
Crystal system	monoclinic
Space group	$P2_1/c$
a, Å	9.9386(9)
b, Å	10.3040(7)
c, Å	11.3175(10)
α, °	90
β, °	114.517(10)
γ, °	90
V, Å^3	1054.51(2)
Z	2
ρ_{calc}, g cm^3	1.463
μ, mm^{-1}	0.468
F(*000*)	480
No. of reflections (unique)	8879 (2565)
S[a]	1.08
$R_1(wR_2)$ ($F^2 > 2\sigma(F^2)$)	0.0661 (0.1380)
R_{int}	0.083
Min./max. diff map, Å^{-3}	-0.35, 0.45

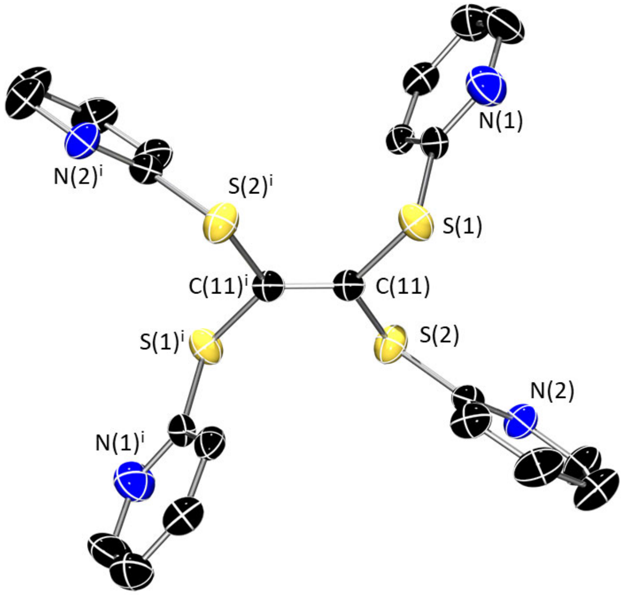

Figure S21: Crystal structure of **3**. Ellipsoids are set at 50% probability level and hydrogens omitted for clarity. The asymmetric unit contains half a molecule; the full molecule has been reproduced here. Symmetry operation used to generate equivalent atoms: i = 1−*x*, 1−*y*, −*z*. C: black; N: blue; S: yellow.

S4. Computational data

Computed Structures (A) and Energies (a. u.)

K(Tptm)

```
BP86 energy = -1111.77927724
Enthalpy 0K = -1111.416953
Enthalpy 298K = -1111.384929
Free energy 298K = -1111.488091
Low freq. = 10.5471 cm-1

C  -1.84675 -0.28620  0.98552
K   0.53346  0.17151 -0.49062
S  -1.45088 -1.08964  2.56875
S  -2.98622 -1.35979  0.06557
S  -2.70476  1.26899  1.35820
C  -2.51910 -1.39498 -1.66901
N  -1.45373 -0.73462 -2.17441
C  -1.24111 -0.81763 -3.51069
C  -3.38964 -2.16358 -2.49043
C  -3.14158 -2.24072 -3.85794
C  -2.03940 -1.54400 -4.39628
H  -0.37048 -0.26026 -3.88478
H  -4.24306 -2.67975 -2.03840
H  -3.79972 -2.83128 -4.50452
H  -1.81243 -1.56400 -5.46582
H   2.04006 -3.87989  4.50680
C   1.72956 -3.32137  3.61710
H   3.47613 -3.86162  2.40467
C   2.53370 -3.30937  2.45712
C   2.07790 -2.56863  1.36466
C   0.52668 -2.62175  3.61461
H   2.66829 -2.53132  0.43811
H  -0.13222 -2.62271  4.48923
N   0.91999 -1.86376  1.34188
C   0.14582 -1.89814  2.44973
H  -1.79520  6.05054  1.51948
C  -1.37424  5.06639  1.28663
H   0.61859  5.80737  0.74910
C  -0.03687  4.93860  0.85629
C   0.42871  3.65374  0.57090
C  -2.15538  3.92190  1.41879
H   1.46295  3.50500  0.22985
H  -3.19378  3.97982  1.76157
N  -0.31312  2.52438  0.68147
C  -1.58958  2.65509  1.10553
H   2.96204 -1.14280 -3.58376
C   3.17611 -0.34244 -2.86749
C   2.75248  0.97313 -3.13226
H   2.21213  1.20466 -4.05598
C   3.05117  1.99228 -2.21186
H   2.73999  3.02274 -2.41367
C   3.76505  1.69559 -1.03645
H   4.00727  2.49900 -0.33094
C   4.19743  0.38188 -0.75613
C   3.89038 -0.63115 -1.69303
H   4.23308 -1.65602 -1.50730
C   4.96984  0.06075  0.50589
H   5.21368  0.97307  1.07306
H   5.91618 -0.45782  0.27319
H   4.39157 -0.60472  1.17193
```

HN(SiMe$_3$)$_2$

```
BP86 energy = -302.752809504
Enthalpy 0K = -302.520538
Enthalpy 298K = -302.502084
Free energy 298K = -302.565282
Low freq. = 20.5758 cm-1

H  -2.76683 -0.22464  2.32229
H   2.78768 -0.24528  2.30964
H  -2.11493 -2.46367  0.18074
C  -2.81723  0.49199  1.48397
C   2.80929 -0.79244  1.35072
C  -2.13772 -1.69575 -0.61109
H   2.23625  2.37929  0.60923
H   2.53817 -1.84220  1.55309
N   0.00244 -0.03384  0.77605
H  -3.86099  0.50569  1.12576
H  -2.58398  1.49546  1.87857
H  -3.15903 -1.66872 -1.03056
Si -1.62902  0.00522  0.07791
H   3.85121 -0.77442  0.98700
H  -1.45379 -2.02152 -1.41306
Si  1.63180 -0.01071  0.07492
C   2.22081  1.76142 -0.30499
H   3.23948  1.76359 -0.73191
H   1.55007  2.25374 -1.02950
C  -1.72503  1.28809 -1.32485
C   1.64440 -1.02151 -1.53605
H   1.36618 -2.07152 -1.34618
H  -2.73597  1.31081 -1.76823
H  -1.49362  2.29903 -0.94959
H  -1.01519  1.05879 -2.13826
H   2.64960 -1.01183 -1.99232
H   0.94212 -0.61299 -2.28289
H   0.00036 -0.00670  1.79814
```

K[N(SiMe$_3$)$_2$]

```
BP86 energy = -602.062506636
Enthalpy 0K = -601.716589
Enthalpy 298K = -601.688171
Free energy 298K = -601.780640
Low freq. = 5.2267 cm-1
```

```
K  -0.82529  0.66218 -0.06430
H   0.90092  2.99138 -0.95139
H  -0.43983 -2.29760  0.86441
H   3.23537  1.52719 -2.46908
C   1.52989  2.94710 -0.03912
C   0.16733 -2.57489 -0.02051
C   3.81265  1.55303 -1.52803
H   2.23532 -2.09226  2.49442
H  -0.39760 -2.31604 -0.93837
N   1.63297 -0.00927 -0.01074
H   2.13649  3.87008 -0.03770
H   0.87166  3.00739  0.85124
H   4.38209  2.50000 -1.50222
Si  2.65660  1.36207 -0.00647
H   0.26479 -3.67498 -0.00694
H   4.54195  0.72509 -1.56917
Si  1.90330 -1.69797  0.01177
C   2.77294 -2.39163  1.57745
H   2.83715 -3.49501  1.56355
H   3.80125 -1.99777  1.65826
C   3.76360  1.58113  1.54744
C   2.84589 -2.42248 -1.49659
H   2.35361 -2.14016 -2.44389
H   4.33111  2.52931  1.52407
H   3.15701  1.56871  2.47013
H   4.49348  0.75630  1.62494
H   2.90672 -3.52547 -1.45830
H   3.87789 -2.03188 -1.53534
H  -4.38376 -2.79483  0.79611
H  -2.82795 -2.73881 -0.06285
H  -3.82736 -0.73622  2.15032
C  -3.86554 -2.35530 -0.07172
H  -4.34781 -2.73127 -0.98840
C  -3.84581 -0.15823  1.21905
C  -3.88679 -0.84290 -0.01747
H  -3.84206  1.75286  2.24537
C  -3.85311  1.24714  1.27381
C  -3.92988 -0.07025 -1.20039
H  -3.97863 -0.57919 -2.17011
C  -3.89897  2.00189  0.08662
C  -3.93738  1.33583 -1.15235
H  -3.92618  3.09565  0.12756
H  -3.99342  1.91081 -2.08301
```

HTptm

BP86 energy = -812.445497420
Enthalpy 0K = -812.195852
Enthalpy 298K = -812.174693
Free energy 298K = -812.248951
Low freq. = 16.7175 cm-1

```
C  -0.01357  0.12498  0.11490
S   0.09984  1.82639 -0.57500
S  -1.23270 -0.05755  1.47794
S  -0.21102 -1.09783 -1.25564
C  -2.75236  0.43015  0.66717
N  -2.68647  0.88614 -0.59523
C  -3.84215  1.25327 -1.19117
C  -3.96154  0.31765  1.39201
C  -5.14383  0.70755  0.75849
C  -5.09171  1.18627 -0.56426
H  -3.74691  1.61802 -2.22124
H  -3.96297 -0.06678  2.41682
H  -6.09929  0.63569  1.28866
H  -5.99625  1.49793 -1.09397
H   4.04612  4.75373 -0.61058
C   3.63568  3.78711 -0.29983
H   5.47370  3.07912  0.65202
C   4.43193  2.86144  0.40055
C   3.85006  1.64309  0.76893
C   2.31103  3.46115 -0.59960
H   4.42660  0.88608  1.31507
H   1.66331  4.15481 -1.14562
N   2.57101  1.31159  0.48997
C   1.82309  2.20147 -0.17869
H   1.44249 -5.72192 -1.39362
C   1.37671 -4.79581 -0.81273
H   2.49420 -5.55159  0.90926
C   1.96273 -4.70579  0.46402
C   1.84322 -3.49524  1.15635
C   0.70511 -3.68882 -1.33564
H   2.28094 -3.37511  2.15546
H   0.23527 -3.72145 -2.32401
N   1.19918 -2.41176  0.67026
C   0.64817 -2.51265 -0.55011
H   0.96361 -0.08139  0.59755
```

$(C_5H_4N\text{-}S)_2C{=}C(C_5H_4N\text{-}S)_2$

BP86 energy = -1107.83579906
Enthalpy 0K = -1107.513733
Enthalpy 298K = -1107.485033
Free energy 298K = -1107.577704
Low freq. = 14.7749 cm-1

```
C  -0.63825 -0.31704  0.08385
S  -1.69759 -0.16142 -1.35482
S  -1.42851 -0.48084  1.69462
H  -5.77544 -2.74074 -2.30061
C  -4.83792 -2.69078 -1.73695
H  -5.10052 -4.61156 -0.72826
C  -4.46911 -3.72873 -0.86298
C  -3.26412 -3.60213 -0.15854
C  -3.99943 -1.57929 -1.86754
H  -2.93772 -4.38164  0.54092
```

```
H  -4.26222 -0.73880 -2.51774
N  -2.42847 -2.54977 -0.28671
C  -2.79984 -1.56683 -1.12215
H  -0.65390  4.47937  1.79337
C  -1.02723  3.57729  2.29057
H  -1.67106  4.60343  4.10980
C  -1.59051  3.65259  3.57501
C  -2.05434  2.46438  4.15861
C  -0.94059  2.33967  1.63872
H  -2.50719  2.46956  5.15837
H  -0.51137  2.25242  0.63389
N  -1.98329  1.26052  3.55727
C  -1.43287  1.21254  2.33024
H  -0.56742  4.13432 -1.63081
H   0.52032  4.94572 -3.74913
C   0.13437  3.49551 -2.18151
C   0.73860  3.94751 -3.35960
N   0.35121  2.27325 -1.64410
C   1.62994  3.07965 -4.01787
H   6.14177 -2.02954  2.30411
H   4.32698 -0.31419  2.62735
C   5.23157 -2.06493  1.69640
H   2.13165  3.38820 -4.94109
C   4.22633 -1.11190  1.88460
C   1.20237  1.45679 -2.28488
C   1.87565  1.81597 -3.47696
C   5.06254 -3.05644  0.71304
C   3.06917 -1.20659  1.07941
S   1.75561 -0.01711  1.38604
H   5.82714 -3.81762  0.53390
C   3.88060 -3.04485 -0.03926
S   1.53646 -0.20715 -1.66909
N   2.88841 -2.14656  0.13861
H   2.57023  1.11752 -3.95467
C   0.72203 -0.23244 -0.06253
H   3.70442 -3.79407 -0.82102
```

K(C_5H_4N-S)

```
BP86 energy = -557.881036737
Enthalpy 0K = -557.678612
Enthalpy 298K = -557.661749
Free energy 298K = -557.726902
Low freq. = 11.8273 cm-1

K  -0.53014 -0.70696 -1.17921
S   1.24020  1.73640 -0.89067
H   5.53491  0.24907  1.02115
C   4.54082 -0.05255  0.67065
H   4.81618 -2.19390  1.07483
C   4.15106 -1.40878  0.70433
C   2.87010 -1.71569  0.24123
C   3.65266  0.90298  0.18735
H   2.52025 -2.75877  0.24771
H   3.92179  1.96180  0.14602
N   1.98518 -0.80805 -0.23416
C   2.35510  0.51837 -0.27303
C  -3.70679 -1.48447 -0.23129
C  -3.86183 -0.23399 -0.85937
C  -3.28837  0.91871 -0.29363
C  -2.97964 -1.56411  0.97112
C  -2.54872  0.85575  0.91057
C  -2.40967 -0.40684  1.53216
H  -4.44616 -0.15266 -1.78274
H  -4.17066 -2.37995 -0.65834
H  -2.86999 -2.52628  1.48358
H  -3.41862  1.88818 -0.78792
H  -1.84965 -0.47910  2.47147
C  -1.87893  2.08480  1.47779
H  -2.46174  2.99600  1.26529
H  -0.87627  2.21354  1.01820
H  -1.74155  2.00334  2.56821
```
